# Histone demethylase KDM4D promotes gastrointestinal stromal tumor progression through HIF1β/VEGFA signalling

**DOI:** 10.1186/s12943-018-0861-6

**Published:** 2018-07-30

**Authors:** Fuqing Hu, Haijie Li, Lu Liu, Feng Xu, Senyan Lai, Xuelai Luo, Junbo Hu, Xi Yang

**Affiliations:** Cancer Research Institute, Tongji Hospital, Huazhong University of Science and Technology, Wuhan, China

**Keywords:** GIST - KDM4D, Proliferation, Migration, Angiogenesis

## Abstract

**Background:**

Gastrointestinal stromal tumour (GIST) is the most common soft tissue sarcoma. The identification of the molecular mechanisms regulating GIST progression is vital for its treatment and prevention. Increasing reports have demonstrated that epigenetic alterations play critical roles in GIST development. However, the role of the histone demethylase KDM4D in GIST progression is poorly understood.

**Methods:**

In clinically matched GIST tissues, KDM4D protein levels were measured by Western blot and immunohistochemical (IHC) staining. KDM4D mRNA levels were examined by quantitative real-time PCR (qRT-PCR). Bioinformatics analysis was used to examine KDM4D expression. The biological effects of KDM4D were investigated in vitro using CCK-8, BrdU/PI, wound healing, colony formation, tube formation and Transwell assays and in vivo using a xenograft mice model. Luciferase assays were used to assess regulation of HIF1β gene promoter activity by KDM4D. ChIP assays were performed to assess KDM4D, H3K36me3 and H3K9me3 occupancy on the HIF1β gene promoter.

**Results:**

We observed a significant upregulation of KDM4D in GIST tissue compared with matched normal tissue and further explored the oncogenic function of KDM4D both in vitro and in vivo. Furthermore, we demonstrated that KDM4D directly interacted with the HIF1β gene promoter and regulated its activity, promoting tumour angiogenesis and GIST progression both in vitro and in vivo. Finally, we demonstrated that KDM4D transcriptionally activates HIF1β expression via H3K9me3 and H3K36me3 demethylation at the promoter region.

**Conclusions:**

Our findings reveal the important roles of the KDM4D/HIF1β/VEGFA signalling pathway in GIST progression, and this pathway may act as a potential therapeutic target for GIST patients.

**Electronic supplementary material:**

The online version of this article (10.1186/s12943-018-0861-6) contains supplementary material, which is available to authorized users.

## Background

Gastrointestinal stromal tumour (GIST) is the most common soft tissue sarcoma and often localizes to the gastrointestinal tract [1, 2]. Currently, the majority of studies indicate that GISTs originate from the mesenchymal pacemaker cells of the gastrointestinal tract known as the interstitial cells of Cajal (ICCs) that harbour multi-oncogenic mutations, such as KIT and PDGFRA [3, 4]. Increasing evidence has demonstrated that those oncogenes play a critical role in GIST tumourigenesis, proliferation, and metastasis. Given the important role of oncogenes in GIST progression, molecular targeted drugs (imatinib) have been employed to cure GISTs harbouring mutant KIT or PDGFRA [5]. Although targeted drugs have revolutionized the treatment of GIST, a significant number of GIST patients experience recurrence within two years due to resistance [6, 7]. In addition, there is no promising treatment for wild-type KIT/PDGFRA GISTs [8]. Thus, to develop novel therapeutic strategies, further understanding of the molecular mechanisms of GISTs is crucial.

Recently, numerous studies have implied that epigenetic alterations play critical roles in a wide range of tumours [9, 10]. Previous studies have also demonstrated that epigenetic alterations are responsible for GIST development [11]. Both DNA hypomethylation and DNA hypermethylation are reported to be closely related to GIST progression. Igarashi S. reported that LINE-1 methylation was associated with malignant GIST profiles and poor prognosis. In addition, more genes are methylated in advanced GIST compared with benign GIST [12]. More important, DNA methylation is associated with aggressive clinical characteristics, strongly indicating that DNA methylation is involved in GIST progression and may act as a novel treatment approach for GIST patients [13]. In addition to DNA methylation, histone methylation is another major epigenetic modification that is a reversible process. Previous studies have implied that changes in histone methylation could lead to gene activation or repression and effect tumour progression [14, 15]. In GIST, histone H2AX is a direct mediator of gastrointestinal stromal tumour cell apoptosis upon treatment with imatinib mesylate [16]. Histones can be modified by methylation and demethylation. Numerous demethylases are involved in diverse tumour development [17]. For example, KDM4 family members demethylate different sites of histones to activate or suppress gene expression [18–20]. However, the potential role of demethylases in GIST remains largely unknown. Importantly, the molecular mechanisms by which demethylases regulate GIST progression remain unclear.

Herein, we demonstrate that KDM4D mRNA and protein levels are upregulated in GIST compared with the matched normal tissues. Further, KDM4D overexpression strongly promotes GIST cell proliferation, migration, invasion and tumour angiogenesis. In contrast, silencing KDM4D reduced cell proliferation, migration, invasion and tumour angiogenesis. More importantly, these biologic effects mediated by KDM4D might be dependent on the Hif1β/VEGFA signalling pathway. These findings highlight novel evidence for a functional link between demethylase and GIST development. We believe that the KDM4D/Hif1β/VEGFA signalling pathway may act as a potential therapeutic target for GIST patients.

## Methods

### Cell lines and regents

GIST882 cells, a kind gift of Jonathan Fletcher (Dana-Farber Cancer Institute, Boston, MA), were derived from a GIST patient with a homozygous missense mutation in KIT exon 13 (K642E). GIST-T1 cells obtained from Biowit Technologies (Shenzhen, China) were derived from a GIST patient with an in-frame deletion of 57 nucleotides in KIT exon 11 (V560Y579del). Both cell lines used in this study were cultured at 37 °C with 5% CO_2_ in DMEM supplemented with 1% penicillin-streptomycin and 10% fetal bovine serum. Antibodies to KDM4D (ab93694) and VEGFA (ab1316) were purchased from Abcam (Cambridge, MA, USA). HIF1a (#36169), H3K36me3 (#4909) and H3K9me3 (#13969) antibodies were obtained from Cell Signaling Technology (Danvers, MA, USA). Antibodies to GAPDH (sc-47,724), HIF1β (sc-17,811), Histone3 (sc-517,576) and mouse IgG (sc-69,786) were purchased from Santa Cruz Biotechnology (Santa Cruz, CA, USA). The human VEGFA ELISA Kit (EK0539) was obtained from Boster Biological Technology (Wuhan, China).

### Immunohistochemistry

Human GISTs samples were obtained from Tongji Hospital. For immunohistochemistry, formalin-fixed paraffin-embedded tissues were cut into 4-mm sections. Then, slides were chosen to stain with antibodies against KDM4D, VEGFA and CD31, separately. Two experienced pathologists examined immunostaining results. The intensity stained scores were evaluated according to the intensity of the nucleic or cytoplasmic staining (no staining = 0, weak staining = 1, moderate staining = 2, strong staining = 3). Finally, the IHC score was generated by combining the intensity scores with the percentage of positively stained cells.

### Quantitative real-time PCR (qRT-PCR)

Total RNA was extracted using TRIzol Reagent (Invitrogen) and reverse transcribed using SuperScript II Reverse Transcriptase (Invitrogen) according to the manufacturer’s instructions. Quantitative PCR was performed using the ABI 7300 real-time PCR system (Applied Biosystems) with KDM4D and GAPDH specific primers. The fold-change in KDM4D expression was calculated using the 2^-△△CT^ method, and GAPDH mRNA levels served as the control. The sense primer for KDM4D was 5’-GGGCAGGGGTGTTTACTCAAT-3′, the antisense primer for KDM4D was 5’-TGTTTGCCAAATGGCGATACT-3′. The sense primer for GAPDH was 5’-ACCACAGTCCATGCCATCAC-3′, the antisense primer for GAPDH was 5’-TCCACCACCCTGTTGCTG TA-3′.

### Western blot

Total cell protein was isolated using NP40 lysates with protease and phosphatase inhibitors. Protein samples were separated by SDS-PAGE and then transferred to PVDF membranes. Blots were incubated with primary antibodies overnight at 4 °C. Then, blots were incubated with the corresponding HRP-conjugated secondary antibodies. Immunoreactive bands were examined with ECL regents (Thermo Scientific).

### Cell viability assay

Cells were seeded into 96-well plates. Overnight, the medium was exchanged with 100 μl of medium supplemented with 10 μl CCK8. The plates were incubated for 2 h. Afterwards, absorbance at 450 nm was measured to obtain OD value. Similarly, other time points (24, 48, 72 and 96 h) were also assessed according to the procedures.

### Soft agar colony formation assay

Briefly, 1% noble agar was added to the bottom of 6-well plates. After the layer of agar solidified, 500 cells were seeded into the 6-well plates. The time required for adequate colony formation varies for each cell line and is typically approximately two weeks. The medium was replaced every three days. After approximately two weeks, photographs of colonies were obtained using a microscope.

### ChIP assay

Chromatin immunoprecipitation assays were performed as described previously [20]. The primers used for CHIP assays are provided below: HIF1β upstream sense, 5’-CGCTCTTGTTGCCCAGACTGG-3′; HIF1β downstream sense, 5’-TCTGTAATCCCAGCACTTTGGG-3′.

### Luciferase reporter assay

Cells were co-transfected with 0.2 μg of HIF1β promoter-luciferase reporter plasmid (PGL3-HIF1β promoter) and 10 ng Renilla luciferase control vector. Firefly luciferase activity was detected using dual-luciferase Reporter Assays (Promega) 48 h after transfection and normalized to Renilla luciferase activity. PGL3-HIF1β promoter was generated using the following primers. The sense primer for the HIF1β promoter was 5’-CTTTGTGATCCGCCCTCCTTG-3′, and the anti-sense primer for the HIF1β promoter was 5’-AGTAGGCGGAGTCAACACAC-3′.

### BrdU/PI assay

BrdU/PI assays were performed as described previously [21]. Briefly, cells were cultured in a 6-well plate overnight followed by a 20-min incubation with 10 μg/ml BrdU. Then, cells were washed twice with PBS and fixed with 70% ethanol at − 20 °C overnight.

Next, cells were denatured in 2 N HCL for 45 min and stained with FITC secondary antibody for 1 h at room temperature. Cells were further incubated with 40 g*/*ml RNase A and 200 g*/*ml PI for 30 min and finally analysed by flow cytometry.

### Endothelial cell tube formation assay

GIST cells were treated as indicated overnight in DMEM supplemented without serum. Then, 20,000 HUVECs cells per well incubated with GIST cell-conditioned media were seeded into the Matrigel in 96-well plates. Microscopic images of tube formation were obtained, and the number of branches in the formed HUVEC tubes was assessed.

### Enzyme-linked immunosorbent assay (ELISA)

GISTs cells were treated as indicated overnight in DMEM supplemented without serum. Overnight, cell-conditioned medium was collected for VEGFA-ELISA analysis according to the manufacturer’s instructions. The OD values were obtained based on absorbance at 470 nm.

### Wound healing and Transwell assays

For the wound-healing assay, GISTs cells were seeded on six-well plates. When 95% confluence was achieved, the cell monolayer was gently scratched using a 200-μm sterile plastic pipette tip. Then, the wound was photographed. After 24 h, the healing wound was photographed. For Transwell migration or invasion assays, 4 × 10^4^ cells suspended in medium without serum were seeded in the upper chamber membranes coated without/with Matrigel (BD Biosciences). Then, 600 μl medium with 10% FBS was added to the lower chamber. After 24 h, the underside of the membrane was fixed for 30 min and stained with 0.1% crystal violet. The inner side of the membrane was wiped with a cotton swab. Then, cells were quantified under a microscope.

### Mouse xenograft tumour assay

All mice experiments were approved by the Animal Care and Use Committee of Tongji Hospital. Four-week Balb/c nude mice were obtained from Beijing Huafukang Bioscience, and 12 mice were randomly divided into 2 groups. We resuspended GIST882 cells stably expressing vector or KDM4D in PBS, mixed the cells with Matrigel in a 1:1 ratio and injected the suspension into the subcutaneous right groin tissue of Balb/c nude mice. After these mice developed a visible tumour mass, tumour volumes were calculated as (length×width×width)/2. Thirty days after subcutaneous injection, mice were sacrificed. Of note, for the mouse xenograft tumor model from ShNC cells and ShKDM4D cells, mice were sacrificed after subcutaneous injection 25d.

### Statistical analysis

Statistical analysis was performed using Prism 5.0 (GraphPad Software). Statistical differences between two groups of data were determined using the unpaired two-tailed Student t-test. Data were considered significant when *p* < 0.05.

## Results

### KDM4D expression is elevated in GIST tissues

**Fig. 1:**
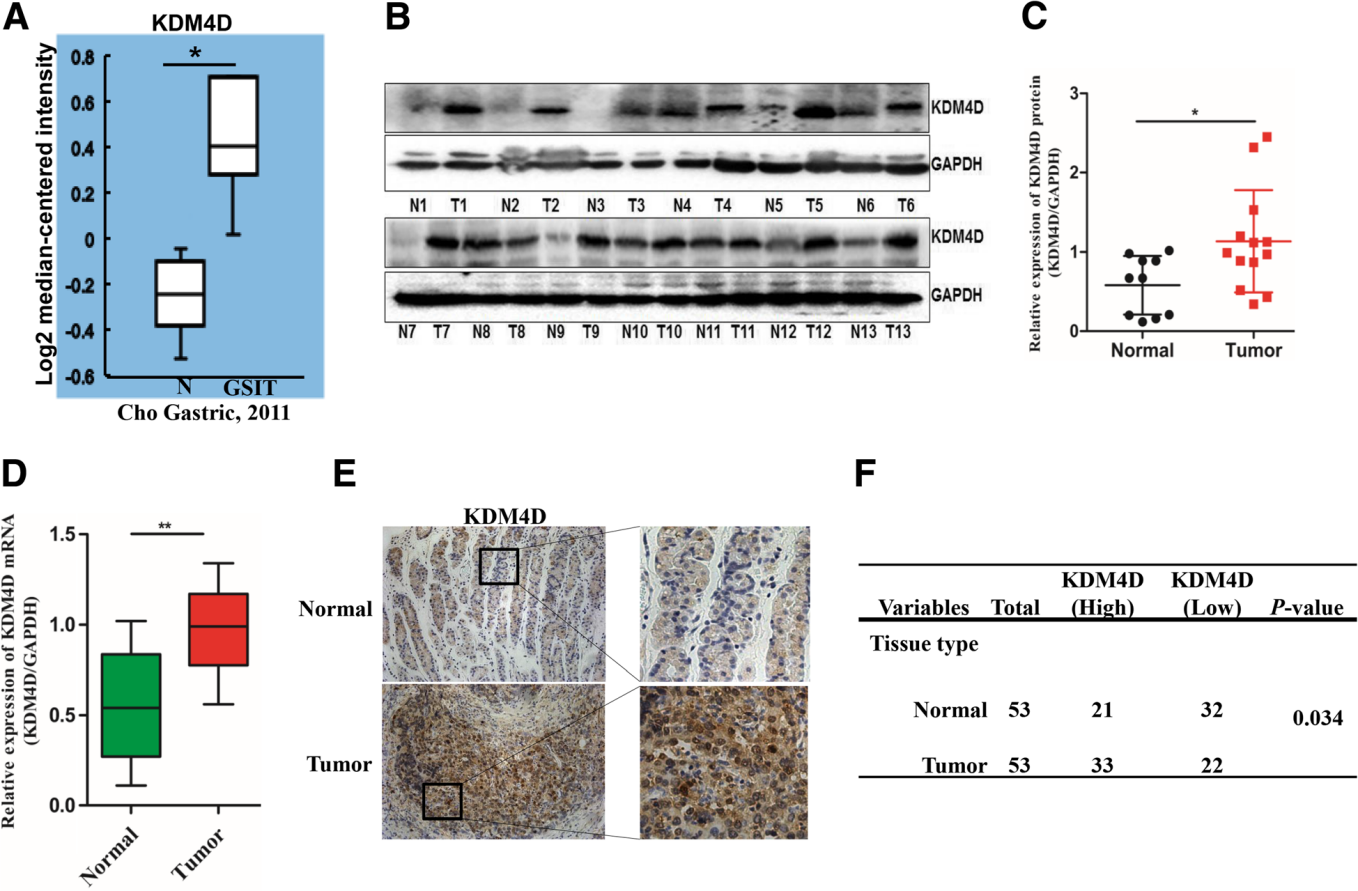
KDM4D expression is elevated in GIST tissue. **a** KDM4D expression was examined in normal and GIST tissues from the Oncomine public data set. **b**, **c** KDM4D protein expression in normal and GIST tissues was analysed by western blot **b**. KDM4D protein expression was quantified using Image J software (**c**). **d** KDM4D mRNA expression was detected in normal and GIST tissues. **e** KDM4D protein expression in normal and GIST tissue was analysed by IHC. **f** The association between KDM4D expression and GIST tissue type was assessed using Pearson’s *χ*^2^ test

To dissect the contribution of demethylases to GIST, we first analysed the data from Oncomine. Surprisingly, we found that KDM4D levels were upregulated in GIST compared with normal tissues. To validate this finding, we further assessed KDM4D expression in our clinical GIST specimens. As shown in Fig. 1b, c and d, elevated KDM4D mRNA and protein expression was observed in GIST samples compared with matched normal tissues. Next, KDM4D expression was detected using IHC. Similarly, IHC results were consistent with prior observations that KDM4D is preferentially overexpressed in GIST samples (Fig. 1e, f). These data indicate that KDM4D may be involved in GIST progression.

### KDM4D promotes GIST cell proliferation, migration and invasion

**Fig. 2:**
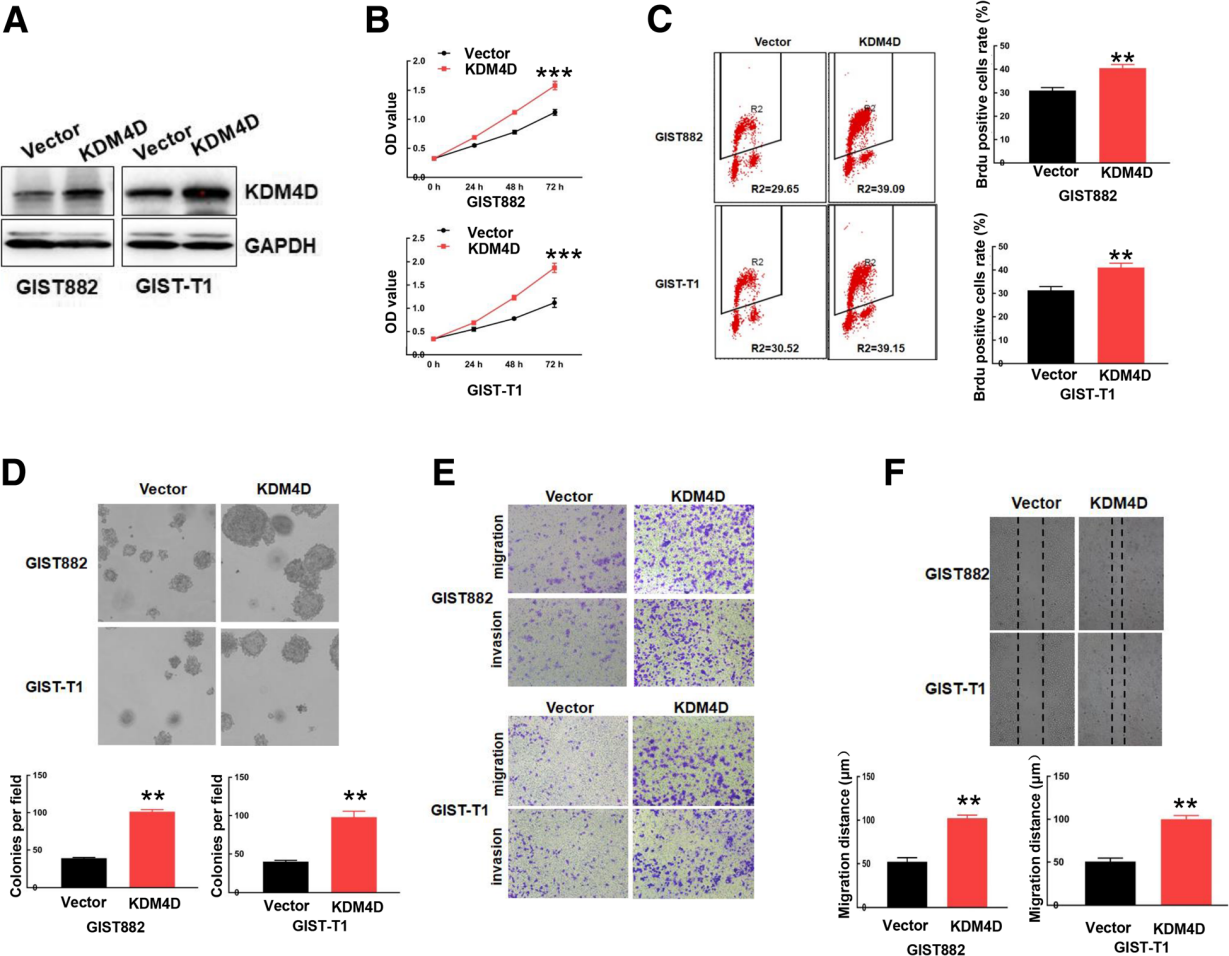
KDM4D promotes GIST cell proliferation, migration and invasion. **a** GIST882 and GIST-T1 cells stably expressing vector or KDM4D were harvested. KDM4D protein expression was examined by western blot, and GAPDH served as the loading control. **b** The viability of GIST882 and GIST-T1 cells stably expressing vector or KDM4D was detected by CCK8 assay. ***p < 0.001 (**c**) DNA synthesis was examined in cells stably expressing vector and cells stably expressing KDM4D using BrdU/PI assays. Quantitative analysis of DNA synthesis is presented in the right panel. **p < 0.01 (**d**) Approximately 500 GIST882 and GIST-T1 cells stably expressing vector or KDM4D were seeded on 6-well plates for soft agar colony formation assays. Quantitative analysis of colony formation is presented in the lower panel. **p < 0.01 (**e**) Both GIST882 and GIST-T1 cells stably expressing vector or KDM4D were plated in the upper chamber with/without Matrigel for 24 h. Then, cell migration and invasion were assessed by observing the cells that migrated to the underside of the Transwell insert. **f** Wound healing assays were used to examine cell migration in GIST882 and GIST-T1 cells stably expressing vector or KDM4D. Quantitative analysis of migration distance is presented in the lower panel. **p < 0.01

Given the abnormal expression of KDM4D in GIST, we then assessed whether KDM4D expression affects GIST progression. To assess this hypothesis, we constructed the pcDNA-3.1(+)-KDM4D plasmid and control vector pcDNA-3.1(+) plasmid. Next, we transfected each plasmid into GIST-882 and GIST-T1 cells to obtain stable cell lines expressing KDM4D or control vector upon treatment with G418. As shown in Fig. 2a, western blot results revealed upregulated KDM4D expression in GIST-882 and GIST-T1 cells expressing pcDNA-3.1(+)-KDM4D compared with vector cells. Further evaluating the role of KDM4D in GIST proliferation, we found, using CCK8 assays, that KDM4D cells exhibit increased growth compared with vector cells (Fig. 2b). Consistent with this result, BrdU/PI assay showed that overexpression of KDM4D increased the number of cells that incorporated BrdU compared with vector cells (Fig. 2b), indicating that KDM4D cells exhibit increased DNA synthesis. Concordantly, KDM4D overexpression also increased the number and size of cell colonies, as determined by soft agar colony formation assays (Fig. 2d). These data indicate that KDM4D overexpression promotes GIST cell proliferation.

Clinically, approximately 20% of GISTs have metastasized at the time of diagnosis. Metastasis seriously limits the treatments available for GISTs. Thus, we further assessed whether KDM4D plays a role in GIST metastasis. As shown in Fig. 2e, overexpression KDM4D markedly increased cell migration in Transwell assays and promoted cell invasion through Matrigel. Consistent with this phenomenon, we observed that KDM4D overexpression strongly increased wound-healing capacity (Fig. 2f). Together, the above data suggest a role of KDM4D in GIST cell motility and invasion.

### Silencing of KDM4D reduces GIST cell proliferation, migration and invasion

**Fig. 3:**
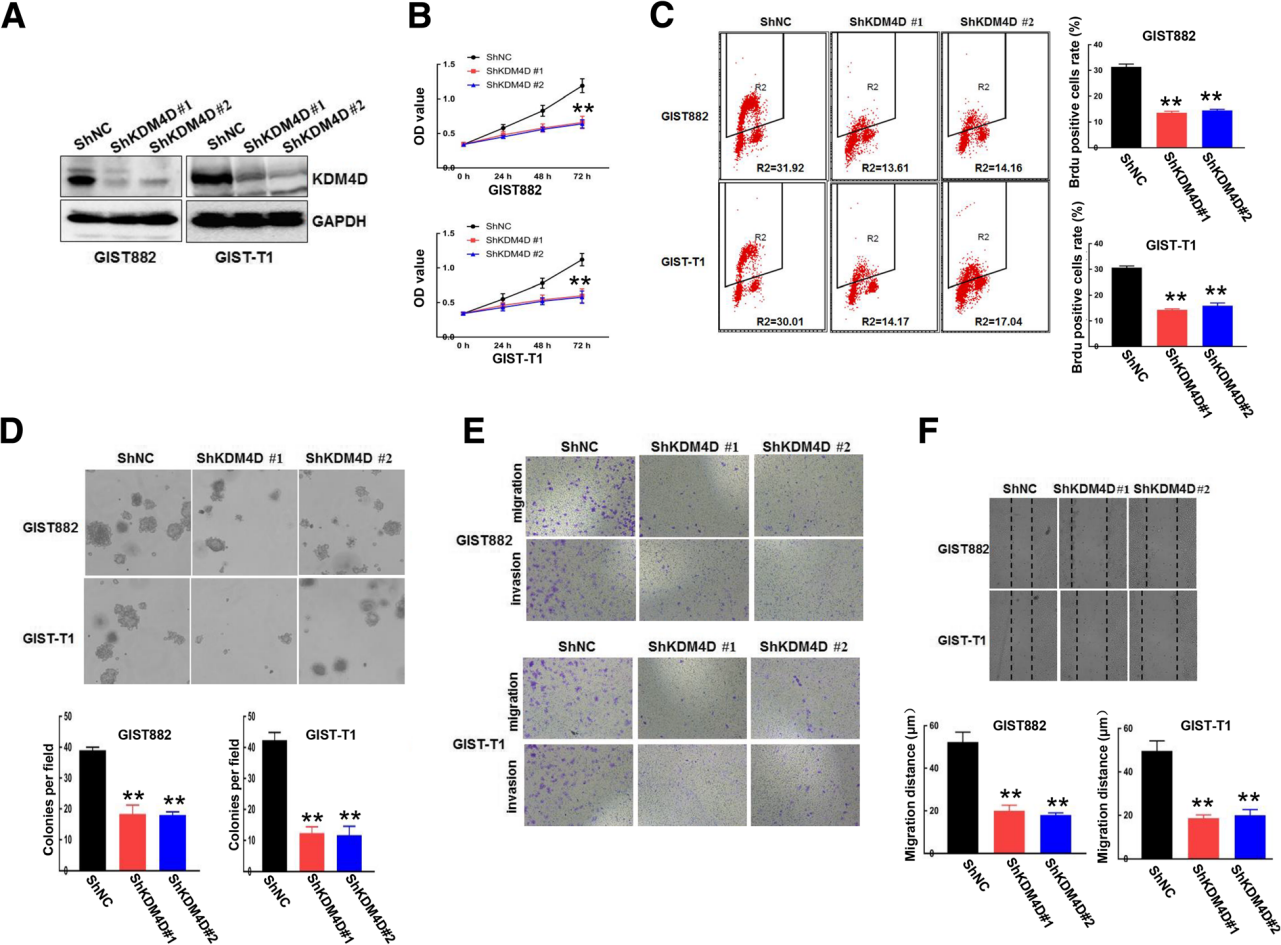
KDM4D silencing reduces GIST cell proliferation, migration and invasion . **a** GIST882 and GIST-T1 cells stably expressing ShNC or ShKDM4D were harvested. KDM4D protein expression was examined by Western blot, and GAPDH served as the loading control. **b** The viability of GIST882 and GIST-T1 cells stably expressing ShNC or ShKDM4D was detected using CCK8 assays. **p < 0.01 (**c**) DNA synthesis ability was examined in cells stably expressing ShNC and cells stably expressing ShKDM4D using BrdU/PI assays. Quantitative analysis of DNA synthesis is presented in the right panel. **p < 0.01 (**d**) Approximately 500 GIST882 and GIST-T1 cells stably expressing ShNC or ShKDM4D were seeded on 6-well plates for soft agar colony formation assays. Quantitative analysis of colony formation is presented in the lower panel. **p < 0.01 (**e**) Both GIST882 and GIST-T1 cells stably expressing ShNC or ShKDM4D were plated in the upper chamber with/without Matrigel for 24 h. Then, cell migration and invasion were assessed by observing the cells that migrated to the underside of the Transwell insert. **f** Wound healing assays were used to examined cell migration in GIST882 and GIST-T1 cells stably expressing ShNC or ShKDM4D. Quantitative analysis of migration distance is presented in the lower panel. **p < 0.01

To better understand the role of KDM4D in GIST proliferation, migration and invasion, we performed lentivirus-mediated knockdown using two independent shRNAs to silence KDM4D expression. Western blot revealed that the shRNAs effectively knocked down KDM4D in sh-KDM4D cells compared with scrambled shRNA (Fig. 3a). As shown in Fig. 3b, KDM4D knockdown strongly decreased GIST cell growth. In addition, KDM4D depletion also reduced DNA synthesis in both GIST-882 and GIST-T1 cells (Fig. 3c). We further found that silencing KDM4D markedly decreased the number and size of cell colonies (Fig. 3d) as determined by soft agar colony formation assays. Next, we assessed the role of KDM4D knockdown in GIST metastasis. Clearly, we observed that KDM4D knockdown markedly abolished cell migration and invasion (Fig. 3e). Consistent with this result, KDM4D knockdown decreased cell mobility in a wound-healing assay (Fig. 3f). Taken together, these data suggest that silencing KDM4D actually inhibits GIST cell proliferation, migration and invasion.

### The emerging role of KDM4D in GIST angiogenesis

**Fig. 4:**
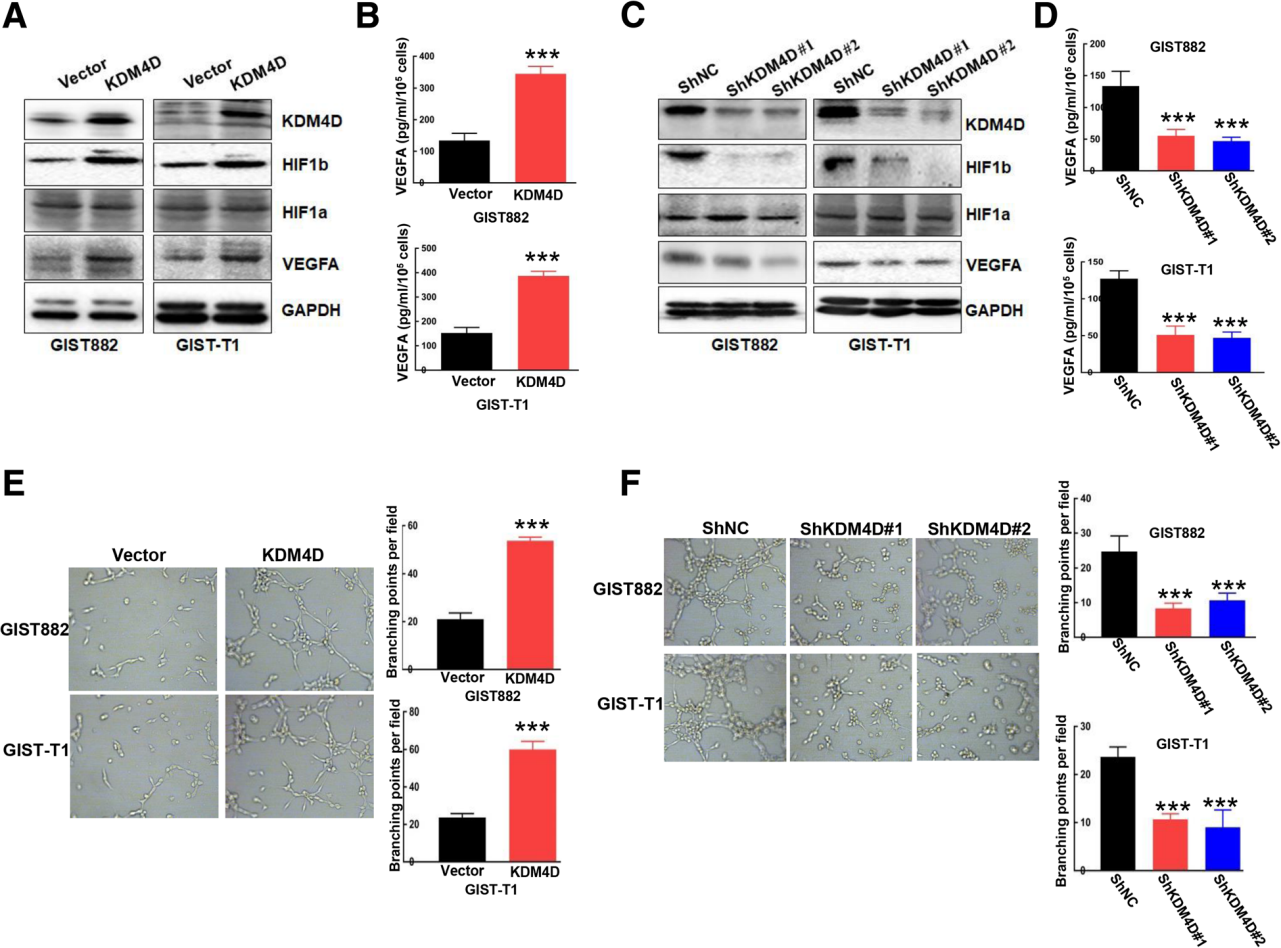
The emerging role of KDM4D in the angiogenic progression of GIST cells. **a** GIST882 and GIST-T1 cells stably expressing vector or KDM4D were harvested, and expression of the indicated proteins was examined by Western blot. GAPDH served as the loading control. **b** GIST882 and GIST-T1 cells stably expressing vector or KDM4D were cultured with DMEM without serum overnight. Conditioned media were collected for ELISA assays to assess VEGFA secretion. ***p < 0.001 (**c**) GIST882 and GIST-T1 cells stably expressing ShNC or ShKDM4D were harvested and expression of the indicated protein was examined by Western blot. GAPDH served as the loading control. **d** GIST882 and GIST-T1 cells stably expressing ShNC or ShKDM4D were cultured with DMEM without serum overnight. Conditioned media were collected for ELISA assays to assess VEGFA secretion. ***p < 0.001 (**e**) Conditioned media from GIST882 and GIST-T1 cells stably expressing vector or KDM4D were incubated with HUVECs cells in 96-well plates. The tube formation was examined by the branching points per filed. ***p < 0.001 (**f**) Conditioned media from GIST882 and GIST-T1 cells stably expressing ShNC or ShKDM4D were incubated with HUVECs cells in 96-well plates. Tube formation was examined based on the number of branching points per field. ***p < 0.001

Angiogenesis, which involves the formation of new capillaries from preexisting microvasculature, plays a vital role in tumour progression. Accumulating data indicate that tumour cells secrete several angiogenic factors (VEGF, PDGFB, and bFGF) to promote angiogenesis [22]. The HIF/VEGFA signalling pathway plays a central role in tumour angiogenesis [23]. Thus, we further evaluate the function of KDM4D in tumour angiogenesis. Interestingly, KDM4D overexpression markedly up-regulated HIF1β expression with minimal effects on HIF1A (which is mainly regulated by hypoxia) (Fig. 4a). In contrast, KDM4D knockdown attenuated HIF1β expression (Fig. 4c). More importantly, elevated VEGFA expression was observed in KDM4D cells compared with control vector cells (Fig. 4a). Consistent with this result, downregulated VEGFA expression was detected in shKDM4D cells compared with shNC cells (Fig. 4c). In addition, KDM4D overexpression increased VEGFA secretion as confirmed using ELISA assays (Fig. 4b). In contrast, silencing KDM4D reduced VEGFA secretion as confirmed using ELISA (Fig. 4d).

To further validate the role of KDM4D in angiogenesis, we cultured endothelial cells incubated with conditioned media (CM) from vector cells, KDM4D cells, shNC cells and shKDM4D cells, separately. As shown in Figs. 4 e and f, we found that CM from overexpression of KDM4D cells robustly induced tube formation of endothelial cells compared with CM from vector cells, and CM from knockdown KDM4D cells significantly decreased tube formation of endothelial cells compared with CM from shNC cells. Collectively, these data strongly suggest that KDM4D potentially influences angiogenesis through the Hif1β/VEGFA signalling pathway.

### HIF1β transcriptional activation by KDM4D correlates closely with demethylation of H3K9me3 and H3K36me3

**Fig. 5:**
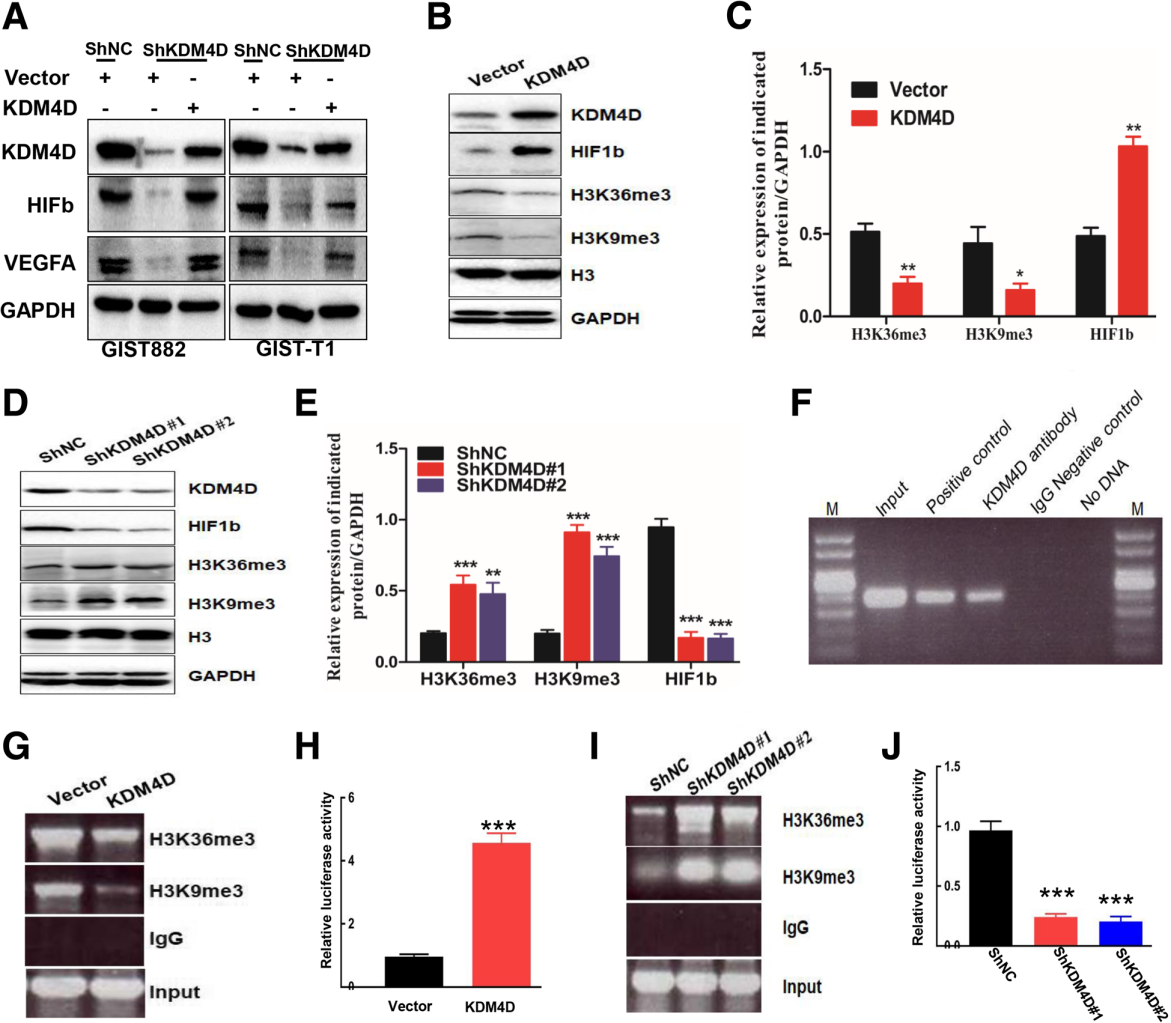
HIF1β transcription activation by KDM4D is closely correlated with H3K9me3 and H3K36me3 demethylation. **a** Western blot reveals HIF1β and VEGFA protein expression among indicated GIST cells. **b**, **c**, **d**, **e** Western blot reveals expression of indicated proteins among vector, KDM4D, ShNC and ShKDM4D GIST882 and GSIT-T1 cells (**b**, **d**); The right histogram showed that the quantification of related protein (c, e). **f** CHIP assay was used to examine the interaction between KDM4D and the HIF1β promoter in GIST882 cells. **g**, **i** CHIP assays were used to examine the interaction between the HIF1β gene promoter and H3K9me3 and H3K36me3 among indicated cells. **h**, **j** Dual-luciferase assays were performed to detect gene luciferase activity of the HIF1β promoter among indicated cells. ***p < 0.001

To further demonstrate whether VEGFA and HIF1β overexpression is a mechanism regulated by KDM4D, we overexpressed KDM4D in shKDM4D cells. Western blot results showed that KDM4D rescue increased VEGFA and HIF1β protein levels (Fig. 5a), indicating that the changes in VEGFA and HIF1β are dependent on KDM4D. Then, we assessed whether KDM4D regulation of GIST phenotypes is dependent on the HIF1β/VEGFA pathway. To assess this hypothesis, we transfected HIF1β plasmids into ShKDM4D cells. CCK8 assays demonstrated that HIF1β overexpression could partially reverse the abolished proliferation mediated by knockdown of KDM4D (Additional file 1: Figure S1A). Furthermore, we found that HIF1β overexpression reverses the attenuated tumour migration, invasion and angiogenesis mediated by silenced KDM4D (Additional file 1: Figure S1B, C). These data strongly indicate that the role of KDM4D in GIST proliferation, migration, invasion and angiogenesis is dependent on HIF1β.

KDM4D is a member of the demethylase family and functions as a demethylase by mainly regulating the methylation levels of H3K36me3. Thus, to explore the mechanism of KDM4D-mediated regulation of HIF1β expression, we first examined methylation levels of histone3 protein. As shown in Figs. 5b, c, d and e, KDM4D overexpression decreased the methylation levels of both H3K36me3 and H3K9me3 compared with vector cells. In contrast, depletion of KDM4D strongly promoted H3K36me3 and H3K9me3 methylation, indicating that KDM4D might bind to the HIF1β gene promoter to regulate its expression. To test this hypothesis, we examined the interaction between KDM4D protein and the HIF1β gene promoter using CHIP assays. As shown in Fig. 5f, the HIF1β gene promoter was directly immunoprecipitated with anti-KDM4D antibodies. Furthermore, CHIP assay results revealed that KDM4D overexpression decreased the interaction between the HIF1β gene promoter and both H3K36me3 and H3K9me3 compared with vector cells and that silencing KDM4D enhanced the interaction (Fig. 5g, i). Next, we further determined whether KDM4D activates the HIF1β gene promoter. Using dual-luciferase assays, we found, interestingly, that HIF1β gene promoter activity was activated in KDM4D cells compared with control vector cells (Fig. 5h). In contrast, KDM4D significantly attenuated Hif1β gene promoter activity compared with shNC cells (Fig. 5j). Taken together, our results demonstrate that KDM4D transcriptionally activates HIF1β expression via demethylation of H3K9me3 and H3K36me3 at the promoter region.

### KDM4D overexpression promotes GIST cell proliferation and angiogenesis in vivo

**Fig. 6:**
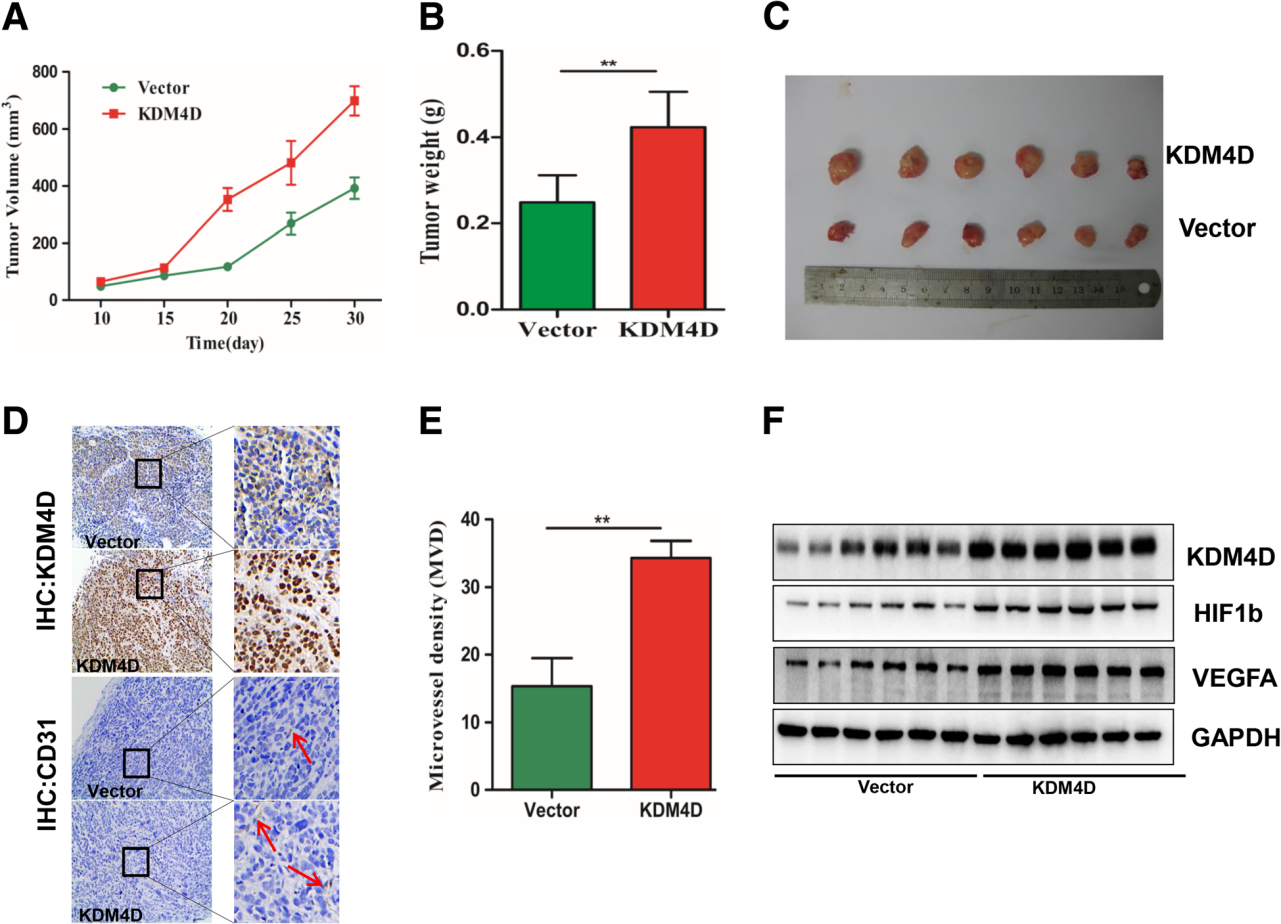
KDM4D overexpression promotes GIST cell proliferation and angiogenesis in vivo. **a** GIST 882 control vector cells or KDM4D cells were subcutaneously injected into Balb/c nude mice. Tumour volume growth curves from day 1 to 30 of treatment are presented. **b** After 30 days, mice were sacrificed, and tumour weights were examined in the two groups. **c** Representative tumour images at the end of the experiment are presented. **d**, **e** Representative IHC staining of KDM4D and CD31 in the two groups is presented. The right histogram presents tumour microvessel density in the groups. **f** Western blot revealed the relationship between KDM4D and VEGFA expression in xenograft tumours

To examine the in vivo pro-tumour activity by KDM4D, vector or stably expressed KDM4D cells were injected into right subcutaneous tissues of Balb/c nude mice. As shown in Fig. 6a, vector cell growth was significantly retarded compared with KDM4D cells in mice. In addition, the weight and size of tumours formed from vector cells were decreased compared with tumours formed from KDM4D cells (Fig. 6b, c). Importantly, immunohistochemistry (IHC) assay results also demonstrated that CD31 expression was significantly upregulated in the KDM4D group compared with the control vector group, indicating that tumour microvessel density was strongly increased in the KDM4D group (Fig. 6d, e). Consistent with this finding, western blot results also demonstrated VEGFA expression was strongly increased in the KDM4D group compared with the control vector group (Fig. 6f).

### KDM4D knockdown suppresses GIST cell proliferation and angiogenesis in vivo

**Fig. 7:**
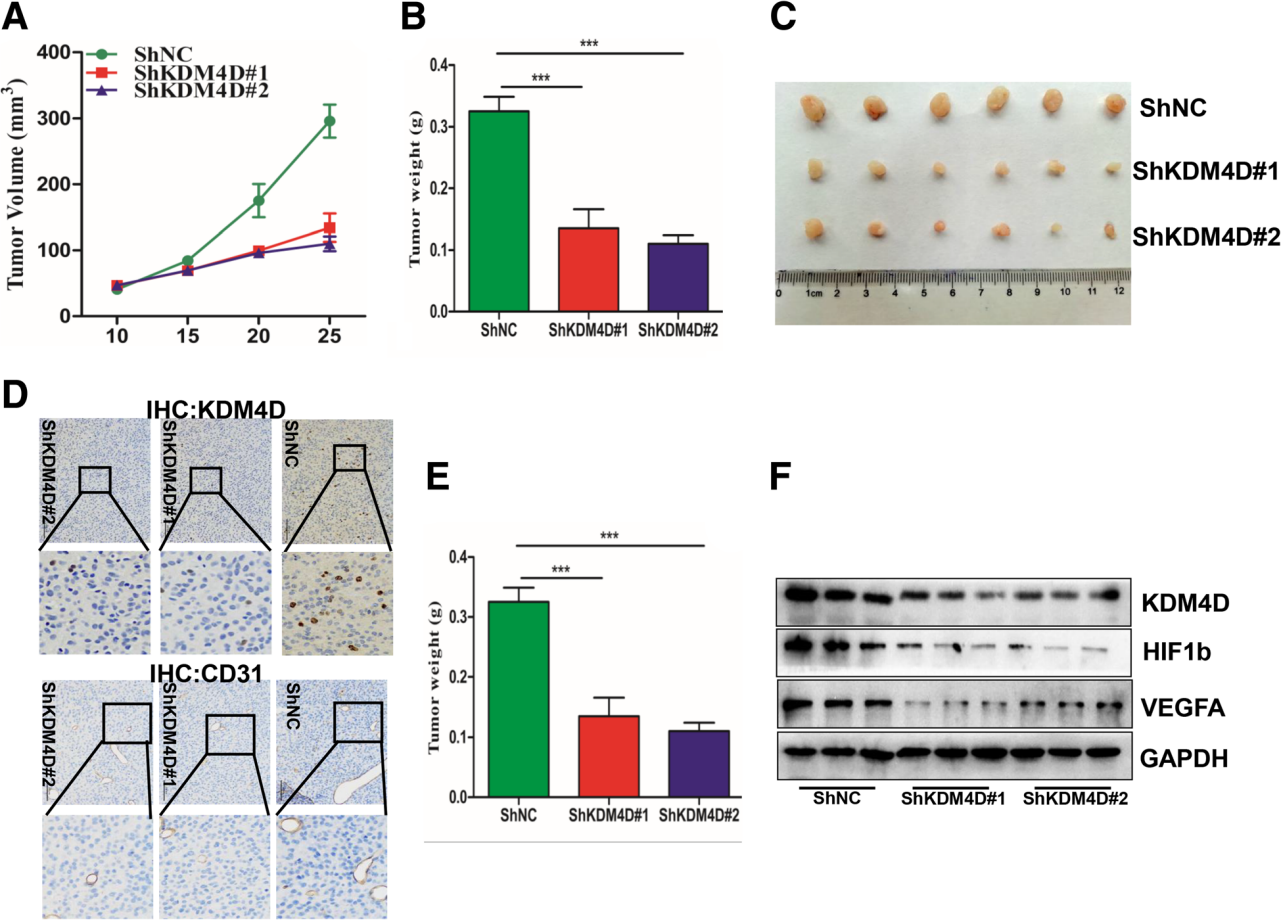
KDM4D knockdown suppresses GIST cell proliferation and angiogenesis in vivo. **a** GIST 882 ShNC cells or ShKDM4D cells were subcutaneously injected into Balb/c nude mice. Tumour volume growth curves from days 1 to 25 of treatment are presented. **b** After 25 days, mice were sacrificed, and tumour weights were examined in the two groups. **c** Representative tumour images at the end of the experiment are presented. **d**, **e** Representative IHC staining of KDM4D and CD31 in the two groups is presented. The right histogram presents tumour microvessel density in the groups. **f** Western blot reveals the relationship between KDM4D and VEGFA expression in xenograft tumours

To further demonstrate the function of KDM4D in GIST proliferation and angiogenesis, ShNC or stably expressing ShKDM4D cells were injected into the right subcutaneous tissues of Balb/c nude mice, separately. In contrast to the overexpression tumour xenograft model, we found, interestingly, that ShNC cells grew significantly faster compared with ShKDM4D cells in mice (Fig. 7a). In addition, the weight and size of tumours formed form ShNC cells were increased compared with tumours formed from ShKDM4D cells (Fig. 7b, c). To assess the status of angiogenesis in xenograft tumours, we examined CD31 expression in the three groups using IHC assays. As shown in Fig. 7d and e, CD31 expression was downregulated in the ShKDM4D group compared with the ShNC group, indicating that the microvessel density was attenuated because KDM4D was silenced in GISTs. Next, we examined the relationship among KDM4D, VEGFA and HIF1β in xenograft mouse models. Western blot results clearly demonstrated that VEGFA and HIF1β expression was strongly decreased in the ShKDM4D group compared with the ShNC group (Fig. 7f). Collectively, these data indicate that KDM4D plays a crucial role in GIST proliferation and angiogenesis.

## Discussion

Most GISTs harbouring mutant KIT or PDGFRA are treated with targeted therapies, such as imatinib. Although limited success is noted at the beginning of treatment, the majority of patients ultimately develop drug resistance [24]. More importantly, wild type GISTs do not respond to molecular targeted therapy [25–29]. Thus, further understanding of the molecular mechanism regulating GIST progression is urgent. Novel treatment strategies should be identified to treat patients with GIST, especially those with unresectable and advanced GIST.

In this study, we found that KDM4D is overexpressed in GISTs compared with corresponding normal tissues. This finding suggests that epigenetic post-translational modifications may be involved in GIST progression. Consistent with our findings, previous data also demonstrated that epigenetic alterations play important roles in GIST development and even have predictive value in GISTs. For example, CD133 methylation percentage is inversely correlated with CD133 protein expression, and hypermethylation in small (< 2 cm) GIST reflects tumour size. Niimura et al. demonstrated that multiple genes in the HOXC cluster are marked by active histone H3K4me3 in malignant GISTs and revealed that HOTAIR might be a potentially useful biomarker in malignant GIST [30]. These data suggested that histone-modifying enzymes could act as novel therapeutic targets in GISTs. KDM4D expression in GISTs has not been previously assessed. Herein, we first evaluated the roles of KDM4D in GIST cell proliferation, migration, and invasion. Surprisingly, KDM4D overexpression strongly increased GIST cell proliferation, migration, and invasion. In contrast, KDM4D depletion significantly attenuated GIST cell proliferation, migration, and invasion, indicating that KDM4D regulated GIST progression. Targeting KDM4D might offer an effective method to control the development of GISTs.

Angiogenesis is a complicated process that is crucial for cancer proliferation and metastasis [31]. Evidence indicates that the Hif/VEGFA signalling pathway is a potent driver of angiogenesis [32]. In the present study, we evaluated the role of KDM4D in tumour angiogenesis. We found that KDM4D overexpression strongly accelerates HUVEC tube formation both in vitro and in vivo. In addition, increased VEGFA secretion was observed in KDM4D cells compared with control vector cells. Consistent with the overexpression experiments, we further found that silencing KDM4D significantly abolished tube formation and decreased VEGFA secretion, indicating that KDM4D regulates GIST development in an angiogenesis-dependent manner. The Hif family plays a critical role in angiogenesis, especially HIF1a, which is mainly regulated by hypoxia. Other Hif members also cooperate with HIF1a to play an important role in tumour progression, including tumour angiogenesis. HIF1β also regulates angiogenesis in a HIF1a-independent manner [33]. To explain the underlying molecular mechanisms of KDM4D-induced angiogenesis, we examined HIF1a and HIF1β protein expression. Interestingly, KDM4D markedly upregulated HIF1β expression but not HIF1a expression.

KDM4D regulates gene expression by regulating the methylation levels of both H3K36me3 and H3K9me3 [34, 35]. Consistent with previous reports, KDM4D overexpression significantly increased H3K36me3 and H3K9me3 methylation levels compared with vector cells. Importantly, CHIP assays revealed that KDM4D interacts with the HIF1β promoter. In addition, changes in KDM4D strongly affect the interaction between the HIF1β promoter and both H3K36me3 and H3K9me3. These findings indicated that KDM4D induces tumour angiogenesis via HIF1β expression. In addition, compared with the control vector group in Balb/c nude mice, the growth of KDM4D-overexpressing cells was increased along with increased VEGFA expression as assessed by immunohistochemistry. These results indicate the pro-tumour effect of KDM4D via tumour angiogenesis.

In summary, our study presents the following lines of evidence supporting the notion that KDM4D plays a vital role in GIST progression. First, KDM4D expression is upregulated in GIST samples. Second, we demonstrated that KDM4D plays a central role in GIST proliferation, migration and invasion both in vitro and in vivo. Third, we demonstrated that KDM4D is required for tumour angiogenesis via regulating VEGFA secretion. Finally, we further revealed that KDM4D directly binds to and activates the HIF1β gene promoter.

## Conclusions

These results identify a novel regulatory pathway (KDM4D/HIF1β/VEGFA) involved in GIST progression. Thus, we propose that elevated KDM4D expression may serve as a biomarker and act as a novel potential target for GIST.

## Additional file

Additional file 1: **Figure S1A.** GIST882 cells stably expressing ShNC or ShKDM4D were transfected with HIF1β plasmids or control plasmids, after transfection for 24h, then, the indicated GIST882 cells were plated into 96-wells. Then, the cell viability of the indicated GIST882 cells was detected at the indicated time by CCK8 assay. **Figure S1B.** Cells were plated in the upper chamber with/without Matrigel for 24 h. Then, cell migrative and invasive ability was assessed by observing the cells migrating to the underside of transwell insert. **Figure S1C.** After transfection for 48h, conditional medium from the indicated GIST882 cells were used to incubated with HUVECs cells in 96 well plates. The tube formation was examined. (TIF 2962 kb)

## Abbreviations

GIST: Gastrointestinal stromal tumour
HIF1β: Aryl hydrocarbon receptor nuclear translocator
IHC staining: Immunohistochemical staining
KDM4D: Lysine-specific demethylase 4D
ShRNA: Short hairpin RNA
VEGFA: Vascular endothelial growth factor A
