# Correction to: Histone demethylase KDM4D promotes gastrointestinal stromal tumor progression through HIF1β/VEGFA signalling

**DOI:** 10.1186/s12943-018-0885-y

**Published:** 2018-09-13

**Authors:** Fuqing Hu, Haijie Li, Lu Liu, Feng Xu, Senyan Lai, Xuelai Luo, Junbo Hu, Xi Yang

**Affiliations:** Cancer Research Institute, Tongji Hospital, Huazhong University of Science and Technology, Wuhan, China

## Correction

After the publication of this work [1] an error was noticed in Fig. 7e, in which the incorrect information is shown. The updated figure (Fig. 7e) included in this correction now shows the quantification of tumor microvessel density. This correction does not affect the findings or conclusions of the article. Nevertheless, we apologize for the inconvenience.

**Fig. 7:**
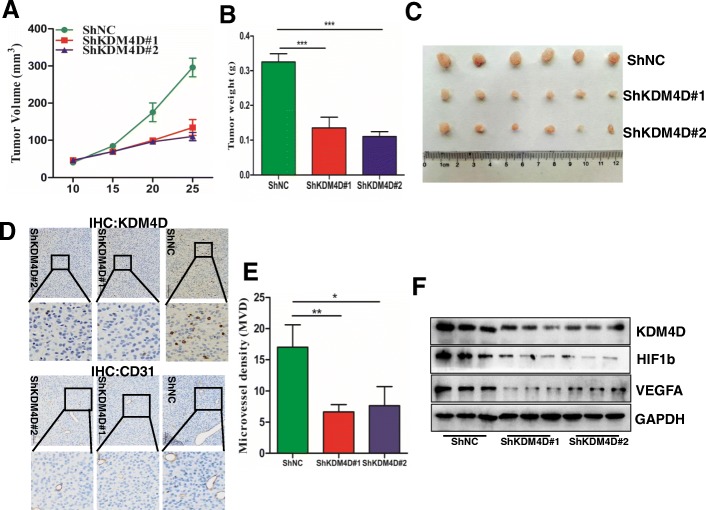
KDM4D knockdown suppresses GIST cell proliferation and angiogenesis in vivo. **a** GIST 882 ShNC cells or ShKDM4D cells were subcutaneously injected into Balb/c nude mice. Tumour volume growth curves from days 1 to 25 of treatment are presented. **b** After 25 days, mice were sacrificed, and tumour weights were examined in the two groups. **c** Representative tumour images at the end of the experiment are presented. **d**, **e**. Representative IHC staining of KDM4D and CD31 in the two groups is presented. The right histogram presents tumour microvessel density in the groups. **f** Western blot reveals the relationship between KDM4D and VEGFA expression in xenograft tumours
