# Supplementary figures and images for: Histone demethylase KDM4D promotes gastrointestinal stromal tumor progression through HIF1β/VEGFA signalling
Source: Mol Cancer. 2018 Jul 30;17:107. doi: 10.1186/s12943-018-0861-6 (PMC6065154; doi:10.1186/s12943-018-0861-6)

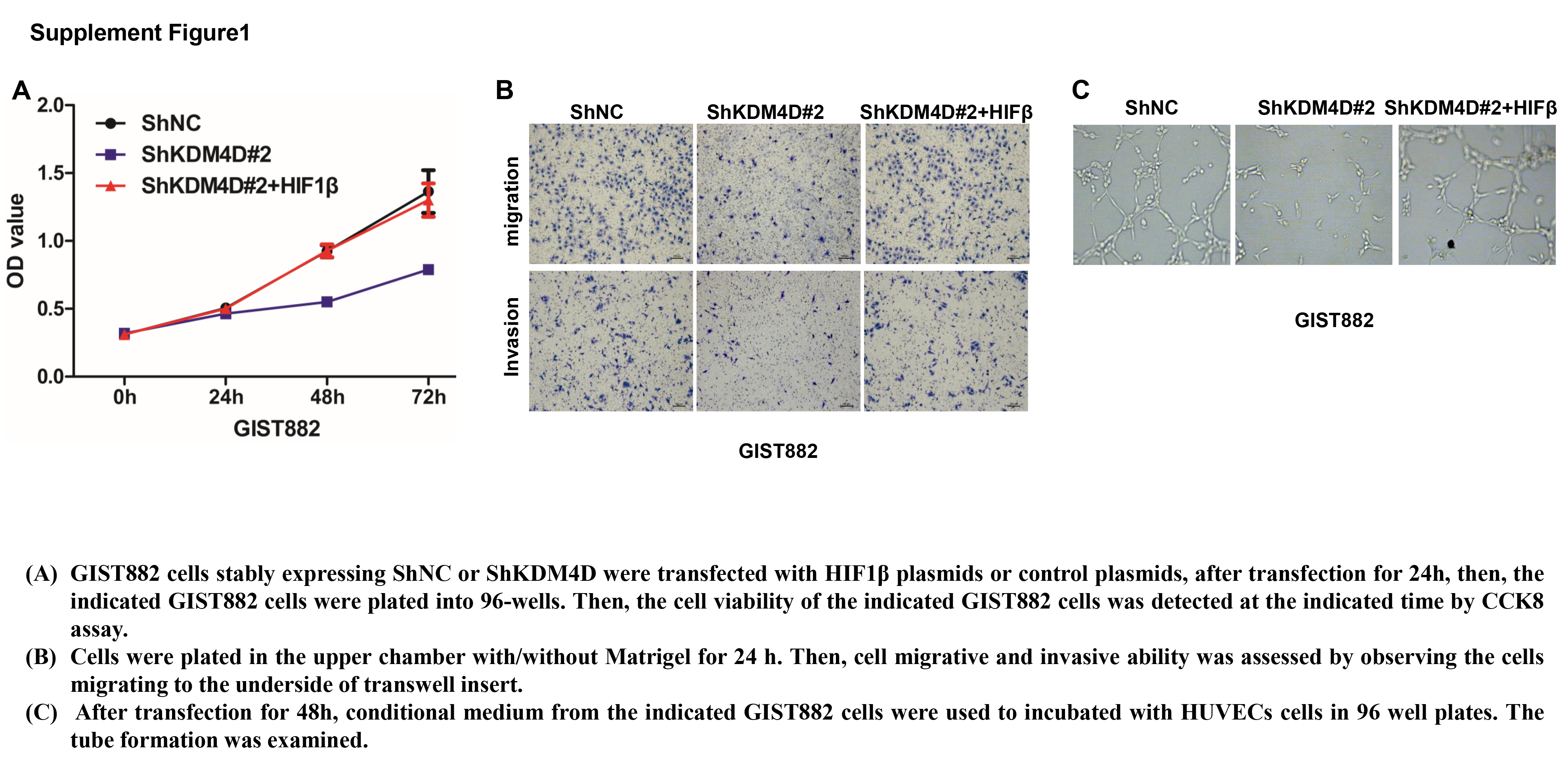

Supplement: Supplementary file 1 — Figure S1A. GIST882 cells stably expressing ShNC or ShKDM4D were transfected with HIF1β plasmids or control plasmids, after transfection for 24h, then, the indicated GIST882 cells were plated into 96-wells. Then, the cell viability of the indicated GIST882 cells was detected at the indicated time by CCK8 assay. Figure S1B. Cells were plated in the upper chamber with/without Matrigel for 24 h. Then, cell migrative and invasive ability was assessed by observing the cells migrating to the underside of transwell insert. Figure S1C. After transfection for 48h, conditional medium from the indicated GIST882 cells were used to incubated with HUVECs cells in 96 well plates. The tube formation was examined. (TIF 2962 kb) [file 12943_2018_861_MOESM1_ESM.tif]
